# Experimental Characterization of an Embossed Capacitive Micromachined Ultrasonic Transducer Cell

**DOI:** 10.3390/mi11020217

**Published:** 2020-02-20

**Authors:** Yuanyu Yu, Jiujiang Wang, Xin Liu, Sio Hang Pun, Shuang Zhang, Ching-Hsiang Cheng, Kin Fong Lei, Mang I Vai, Peng Un Mak

**Affiliations:** 1 Data Recovery Key Laboratory of Sichuan Province, College of Computer Science and AI, Neijiang Normal University, Neijiang 641100, China; cdyu@163.com (Y.Y.);; 2 State Key Laboratory of Analog and Mixed-Signal VLSI, University of Macau, Macau 999078, China; 3 Department of Electrical and Computer Engineering, Faculty of Science and Technology, University of Macau, Macau 999078, China; 4 School of Automotive Engineering, Wuhan University of Technology, Wuhan 430070, China; 5 Graduate Institute of Medical Mechatronics, Chang Gung University, Taoyuan 33302, Taiwan; 6 Department of Radiation Oncology, Chang Gung Memorial Hospital, Linkou 33305, Taiwan

**Keywords:** capacitive micromachined ultrasonic transducer (CMUT), embossed CMUT, collapse mode, output pressure

## Abstract

Capacitive Micromachined Ultrasonic Transducer (CMUT) is a promising ultrasonic transducer in medical diagnosis and therapeutic applications that demand a high output pressure. The concept of a CMUT with an annular embossed pattern on a membrane working in collapse mode is proposed to further improve the output pressure. To evaluate the performance of an embossed CMUT cell, both the embossed and uniform membrane CMUT cells were fabricated in the same die with a customized six-mask sacrificial release process. An annular nickel pattern with the dimension of 3 μm × 2 μm (width × height) was formed on a full top electrode CMUT to realize an embossed CMUT cell. Experimental characterization was carried out with optical, electrical, and acoustic instruments on the embossed and uniform CMUT cells. The embossed CMUT cell achieved 27.1% improvement of output pressure in comparison to the uniform CMUT cell biased at 170 V voltage. The fractional bandwidths of the embossed and uniform CMUT cells were 52.5% and 41.8%, respectively. It substantiated that the embossed pattern should be placed at the vibrating center of the membrane for achieving a higher output pressure. The experimental characterization indicated that the embossed CMUT cell has better operational performance than the uniform CMUT cell in collapse region.

## 1. Introduction

Capacitive Micromachined Ultrasonic Transducer (CMUT) is a promising ultrasonic transducer [1] and has been widely applied in medical imaging [2,3], nondestructive measurement [4], chemical sensing [5], and photoacoustic tomography [6]. CMUT is an electronic transducer based on micro-electro-mechanical system (MEMS) technology so that it owns intrinsic advantages, such as compatible process with integrated circuits, wide bandwidth, and excellent stability of electrical and thermal. However, the low output pressure is still one major limitation of CMUT that restricts its further development in areas dominated by the piezoelectric ultrasonic transducer. As for a CMUT with a uniform membrane, the clamped membrane and the arising spring force from the bending of a uniform membrane restricts the amplitude in vibrating that results in a limited output pressure [7]. In recent years, several solutions have been proposed to alter CMUT structure or the working mode for achieving higher output pressures. Researchers have developed novel CMUT structures to improve the membrane displacement, e.g., making a thicker center on membrane to make it move like a piston [8], using a structure of dual-top-electrode to achieve a higher volume displacement [9], designing an indirectly clamped membrane to enlarge the average diaphragm displacement [10], using multiple moving membranes to increase the amplitude of membrane displacement [11], introducing a structure with substrate-embedded springs to achieve a large average volume membrane displacement [12], proposing an annular cell geometry to enlarge the average membrane displacement [13,14], and forming trenches on the membrane to obtain more displacement [15]. Some other solutions apply a DC bias voltage larger than the pull-in voltage for driving CMUTs to work from the conventional mode to other working modes, for instance, collapse mode [16,17], collapse-snapback mode [18], and deep collapse mode [19]. A method by constructing an annular embossed pattern on the membrane of a collapse mode CMUT to enhance the output pressure has been proposed in our previous works [20,21]. However, the improvement of output pressure brought by the embossed pattern has not been verified on devices yet. In this work, the design and experimental characterization of an embossed CMUT cell is to be presented.

## 2. Design and Fabrication of Embossed CMUT

**Figure 1:**
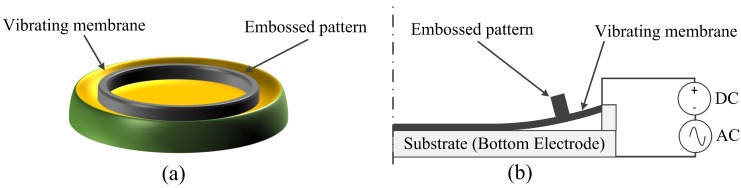
(**a**) Overview of an embossed Capacitive Micromachined Ultrasonic Transducer (CMUT) cell; (**b**) two-dimensional axisymmetric cross sectional view of an embossed CMUT cell in collapse mode.

The concept of an embossed CMUT cell working in collapse mode is described in Figure 1. An annular embossed pattern is attached on a CMUT with a full top electrode. When an exerted DC bias voltage is larger than the pull-in voltage, the high electric field inside the gap will make the membrane collapsed, which is denoted as collapse mode. In collapse mode, the central membrane contacts on the substrate while the outer membrane vibrates to generate ultrasound when superimposing an AC voltage. Similar to the approach of making a thicker center membrane [8], the embossed pattern at the outer vibrating membrane can increase the membrane average displacement and therefore the output pressure is improved. Analyzed with a simply supported beam model in [20], the optimum position of the embossed pattern should be consistent with the vibration center of membrane. Since the contact radius of a collapsed membrane changes with different DC bias voltages, it is a possible way to adjust the membrane vibration center to the embossed pattern. In order to achieve more improvements in output pressure, the embossed pattern was made of nickel for its high density.

### 2.1. Embossed CMUT Design

The dimension of membrane and gap height directly affect the pull-in voltage and center frequency of a CMUT. Based on the fabrication limitations, there are ways to make the uniform and embossed CMUTs with similar pull-in voltages and/or center frequencies. However, as the variations in fabrication processes and misalignments can greatly affect the performances of the CMUT cells working in collapse mode [16], our experiments were designed to minimize these variations. In our current work, all CMUT cells were fabricated on one wafer with the sacrificial release process so that they share the same membrane thickness, gap height, and insulator thickness. Most importantly, all the uniform and embossed CMUTs underwent nearly the same production processes, fabrication variations, and alignment errors in a fair comparison environment, except that the embossed CMUT cells required an additional mask and process for forming the embossed pattern on the membrane. Therefore, in this study, we did not design the uniform and embossed CMUT that have similar pull-in voltages and/or center frequencies. Both the uniform and embossed CMUTs shared the same dimensions except for the embossed pattern.

In this design, both the full top electrode embossed CMUT and the uniform membrane cells were fabricated together for comparison. Firstly, analytical methods were used to estimate the initial design parameters of both CMUT cells. Because the analytical equation for calculating the center frequency of an immersion CMUT working in collapse mode has not been well developed yet, the equation for evaluating the center frequency of a uniform membrane CMUT immersed in liquid was used for initially investigating the relationship of frequency and design parameters, as shown in Equation (1) [22].

(1) $$f_{r}=\frac{\frac{2.98h}{r^{2}}\sqrt{\frac{E}{\rho_{p}\left(1-\sigma^{2}\right)}}}{\sqrt{1+0.67\frac{\rho_{l}r}{\rho_{p}h}}}$$

In this equation, $f_{r}$ is the damped angular resonant frequency of a circular membrane, *h* is the thickness of membrane, *r* is the radius of circular membrane, *E* represents the Young’s modulus of membrane material, σ is the Poisson’s ratio of membrane material, and $\rho_{p}$ and $\rho_{l}$ depict the densities of membrane and liquid, respectively. It can be found that the membrane’s properties and dimensions affect the center frequency of a CMUT cell. A low stress silicon nitride with the material prosperities of E≈220 GPa, σ≈0.263, and $\rho_{p}\approx 3270$ kg/m${}^{3}$ is used for membrane [21]. The liquid medium is corn oil with density of $\rho_{l}\approx 920$ kg/m${}^{3}$ [23]. An explicit equation depicts the relationship of the resonant frequency with a DC bias voltage and pressure was also introduced to analyze the frequency range of the uniform CMUT [24].

Pull-in voltage is also a critical factor that determines the operating point of a CMUT. The pull-in voltage of a uniform membrane CMUT without loads or initial stress can be estimated by Equation (2) [25].

(2) $$V_{pull-in}=\frac{5.369d_{0}}{r^{2}}\sqrt{\frac{Dd_{0}}{\epsilon_{0}}}$$

where *D* is the flexural rigidity of membrane, which is calculated with material prosperities and membrane dimensions, $d_{0}$ is the effective gap height between top and bottom electrodes, $\epsilon_{0}$ is the permittivity of free space.

**Figure 2:**
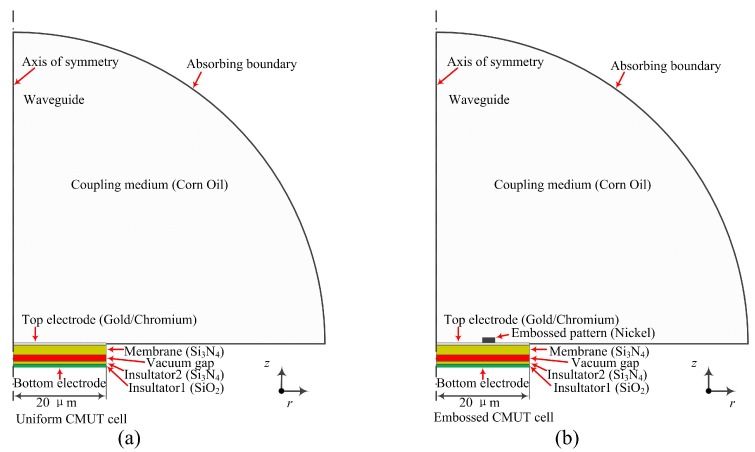
Two-dimensional axisymmetric finite element analysis (FEA) models in COMSOL (Not drawn in scale). (**a**) A uniform CMUT cell; (**b**) An embossed CMUT cell.

With the aforementioned Equations, the CMUT design parameters including membrane dimensions and gap height can be approximately estimated at the initial investigation stage. However, these equations are not suitable for the embossed CMUT working in collapse mode. Therefore, finite element analysis (FEA) assisted with COMSOL Multiphysics 4.4 (COMSOL Inc., Stockholm, Sweden) was applied to finalize design parameters. Both the uniform and embossed membrane CMUT cells were simplified as two-dimensional axisymmetric models to represent the bottom electrode, insulators, vacuum gap, membrane, and embossed pattern, as depicted in Figure 2. Upon each CMUT cell model, a hemispherical waveguide filled with corn oil was built to represent the surrounding media of a CMUT working in immersion. The radius of waveguide was 400 μm which was larger than one wavelength of ultrasound in corn oil and its outer surface was applied with an absorbing boundary to eliminate wave reflection in simulation [26].

The electromehanics (emi) physics in COMSOL was used to govern both the CMUT cell and the waveguide, whereas pressure acoustic (acpr) physics was applied to the waveguide. The maximum mesh size inside the acpr physics was one-eighth of the ultrasound wavelength. In the FEA simulation, non-convergence arises as the membrane contacts with the insulator when applying a DC bias voltage larger than the pull-in voltage. To avoid this problem, a penalty or barrier method was used to deal with the contact process [27]. A prestressed analysis was used to calculate the pressure generated by the CMUT cell over a range of frequency. With this manner, a DC bias voltage was firstly exerted step by step even after the membrane collapsed, then a small AC voltage was superimposed with sweeping frequencies and the surface pressure upon the CMUT cell was calculated by averaging pressures along the interface between the CMUT membrane and medium. By using the FEA models, the design parameters of the embossed membrane CMUT were defined as in Table 1. The nickel embossed pattern was centered at r=12μm with the dimension of 3 μm × 2 μm (width × height) in consideration of our fabrication process ability. The design parameters of the uniform CMUT were the same with the embossed CMUT but with no embossed pattern. The material of membrane was a low stress silicon nitride deposited with low pressure chemical vapor deposition (LPCVD) according to our fabrication process. The residual tensile stress of the membrane was set as 60 MPa. As for the embossed CMUT model, an internal tensile stress of the nickel pattern was set as 370 MPa in consideration of the Watts electroplating method [28]. In addition, the gravity of the embossed pattern was introduced as a load on top of the membrane.

**Figure 3:**
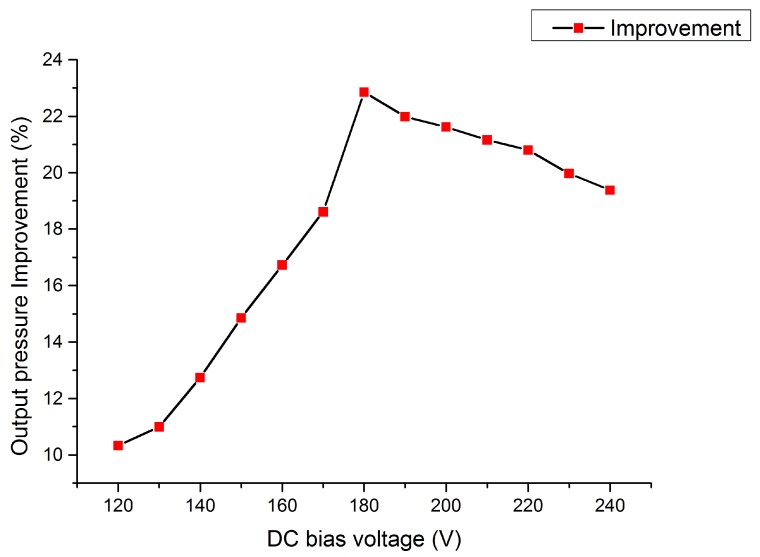
Output pressure improvement of the embossed CMUT cell in FEA simulation.

With the FEA simulation, the estimated pull-in voltages of the embossed and uniform membrane CMUT were 88 V and 122 V, respectively. The additional tensile stress and the gravity of the embossed pattern on the membrane formed a more curved initial gap height that resulted in a lower pull-in voltage of the embossed CMUT than that of the uniform CMUT. Figure 3 showed the output pressure improvement of the embossed CMUT compared with the uniform CMUT in collapse mode and the maximum improvement was about 23% at 180 V.

### 2.2. Embossed CMUT Fabrication

**Figure 4:**
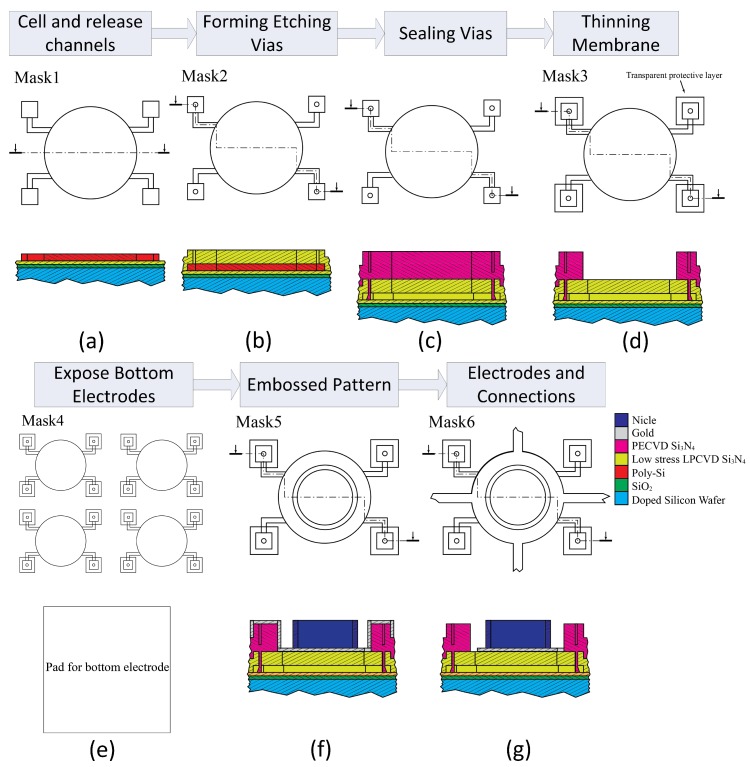
Illustration of the fabrication process flows for the embossed CMUTs. (**a**) Forming a CMUT cell; (**b**) forming etching vias; (**c**) sealing vias; (**d**) thinning the membrane; (**e**) exposing bottom electrodes; (**f**) forming an embossed pattern; (**g**) forming electrodes and connections.

For the sake of comparison, both the embossed and uniform CMUT cells with the same dimensions were fabricated in one die. The CMUTs were fabricated at the Nano Facility Center (NFC) of National Chiao Tung University, Nano-Electro-Mechanical-System (NEMS) Research Center, National Taiwan University, and Bio-MEMS Laboratory of Chang Gung University, Taiwan. Figure 4 illustrates the customized six-mask sacrificial release process for fabricating the embossed CMUTs with top and cross-sectional views from the dashed lines.

In this process, the CMUTs were designed and fabricated on an *n*-type highly doped silicon wafer that also acted as the common bottom electrode of devices. Firstly, a 150-nm SiO_2_ and 150-nm Si_3_N_4_ layers were deposited in sequence with a dry thermal oxidation and a LPCVD process to form a combined insulator layer. Then a 300-nm poly-silicon layer was deposited on the insulator layer as the sacrificial layer. Each cell with four release channels was defined with the Mask #1 and formed on the sacrificial layer by a reactive ion etching (RIE) process, as shown in Figure 4a. Afterwards, the wafer was coated with a 650-nm low stress silicon nitride membrane by LPCVD and four 2-μm vias to the sacrificial layer were formed at the end of release channels by the Mask #2 and a RIE process, as depicted in Figure 4b. In order to construct the cavity, the poly-silicon layer underneath the membrane was fully etched away with potassium hydroxide (KOH) solution. After releasing the sacrificial layer, the wafer was deposited by a 1.2-μm Si_3_N_4_ layer with a plasma-enhanced chemical vapor deposition (PEVCD) process to seal the vias for forming vacuum cavity, as shown in Figure 4c. Therefore, the membrane was thickened by the coated nitride layer and should be etched back to its initial thickness of 650-nm. In the thinning process, the coated nitride layer around the sealed vias should be preserved with the Mask #3 in photolithography and an RIE process, as shown in Figure 4d. Since the whole wafer was coated with multiple insulation layers, these materials in the bottom pad area must be etched before depositing metal. The Mask #4 defined the bottom pad areas with photolithography and the insulation layers in these areas were etched away by a RIE process as depicted in Figure 4e. The wafer was firstly deposited with 20-nm chromium as the adhesion layer and subsequently with 180-nm gold layer as the electrode. The wafer was exposed with the Mask #5 to define the design for the embossed pattern on gold. Nickel patterns were constructed using the Watts nickel electroplating method [29] with a recipe of NiSO4·6-7H_2_:NiCl2·6H_2_O:H_3_BO_3_:H_2_O = 676:114:96:2400 (g) in the condition of 80 m DC current and 48 °C bath temperature. It took 50 s to plate a nickel layer of 2 μm on the wafer, as shown in Figure 4f. In consideration of the total area on the wafer for plating, the calculated current density was about 135 mA/cm${}^{2}$. The final process was to form the top electrodes, bonding pads, and interconnections of CMUT elements by the Mask #6 and using wet etching methods for removing the residual gold and chromium, as depicted in Figure 4g. The details of the whole fabrication process can be found in reference [21].

## 3. Characterization of Embossed CMUT

The CMUT array containing both the uniform and embossed CMUT cells were diced and bonded to external pads on a PCB with aluminum wires for device characterization.

### 3.1. Optical Metrology Characterization

**Figure 5:**
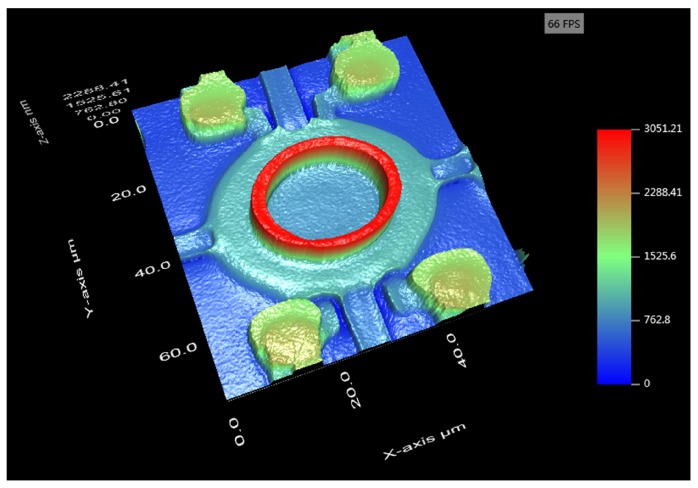
Pseudo-color image of an embossed CMUT (The scale in z-axis was not the same to x-axis and y-axis).

In this study, a 3-D optical microscope (NanoX-2000, ZhenJiang Subnano Instruments Inc., Zhenjiang, China) was employed to measure the vertical and lateral dimensions of the embossed CMUTs. By using a scanning white light on the embossed CMUTs and processing with interference technology and phase shifting interferometry, the 3D surface profile of the embossed CMUT can be precisely measured. The vertical and lateral resolutions of NanoX-2000 are 0.1 nm and 0.48 μm. Figure 5 was the pseudo-color image of an embossed CMUT with an annular embossed pattern, the full top electrode, interconnections, release channels, and sealed vias.

**Figure 6:**
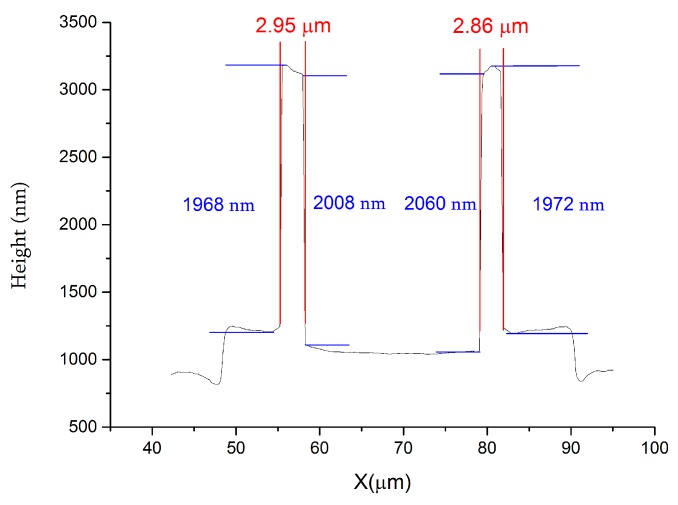
Measured profile of an embossed pattern.

To measure the dimensions of the nickel embossed pattern, a 2-D profile was plotted in Figure 6. The width and height of the annual embossed pattern was approximate 2.9 μm and 2.0 μm, which were close to the design parameters. It can be found that the top surface of the embossed pattern was tilted because the nickel was electroplated on a deflected membrane under an atmospheric pressure.

### 3.2. Electrical Characterization

**Figure 7:**
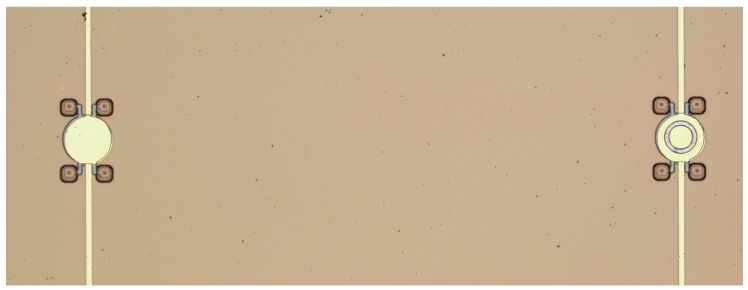
A uniform CMUT cell (**left**) and an embossed CMUT cell (**right**).

The electrical input impedances of both the uniform (**left**) and embossed (**right**) CMUT cells arranged in Figure 7 were measured by an impedance analyzer (Model: 4395A, Agilent Technologies Inc., Santa Clara, CA, USA) with an RF impedance adapter (Model: 43961A, Agilent Technologies Inc., Santa Clara, CA, USA) and a spring clip fixture (Model: 16092A, Agilent Technologies Inc., Santa Clara, CA, USA). A programmable DC power supply (Model: HSPY-400-01, Beijing HanShengPuYuan Technology Co., Ltd., Beijing, China) was used for proving bias voltage.

As for CMUTs working in collapse mode, a charging effect is a common issue as the high electric fields can force charges into insulator materials. In order to reduce the charging effects in measurements, a step DC bias voltage was used instead of applying the DC bias gradually. In addition, after each measurement, the DC power supply was shut down for several minutes before the next measurement.

The resonant frequencies of CMUT cells under various DC bias voltages were measured with the input impedance measurement results, from which the pull-in voltages can also be estimated. The pull-in voltages of the embossed and uniform CMUT cells were about 55 V and 110 V, respectively. The pull-in voltages were lower than the simulation results and it might be ascribed to the deviations of membrane thickness control in fabrication.

It was also found that the embossed CMUT cell owned a much lower pull-in voltage that might be caused by several reasons. According to reference [30], a high current density in nickel plating produces a high tensile stress in the nickel layer. In our fabrication, the low stress silicon nitride membrane deposited by LPCVD also yielded a tensile stress. The overlapped embossed pattern would enlarge the effective tensile stress of the combined layers that resulted in more deflections towards the insulator and formed a smaller initial gap height. In addition, it was mentioned that the modulus of elasticity of nickel decreases linearly on increasing the current density in the reference [30]. In this work, the high current density of 135 mA/cm${}^{2}$ in electroplating might produce the embossed pattern with a high tensile stress and a lower modulus of elasticity, which lead to a much lower pull-in voltage than that in FEA simulation.

**Figure 8:**
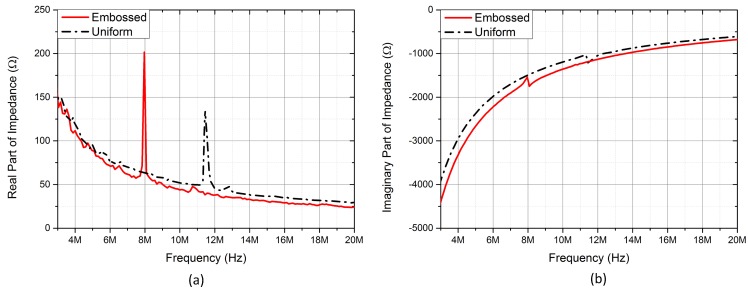
Electrical input impedance of the embossed and uniform CMUT cells bias at 150 V. (**a**) Real parts; (**b**) imaginary Parts.

Figure 8 depicted the electrical input impedances in real and imaginary parts of the embossed and uniform CMUT cells biased at 150 V, which was higher than the pull-in voltages of both devices. The resonant frequency of the embossed CMUT was about 8 MHz that was lower than 11.4 MHz of the uniform CMUT because the attached embossed pattern decreased the elasticity of membrane.

### 3.3. Acoustic Characterization

In this work, a calibrated hydrophone (Model: HGL-200, ONDA Co., Sunnyvale, CA, USA) connected with a 20-dB preamplifier (Model: AG-2020, ONDA Co., Sunnyvale, CA, USA) was used to measure output pressures of the embossed and uniform CMUT cells bonded on a PCB. Both the CMUTs were immersed in a box filled with corn oil for providing electrical insulation and the hydrophone was attached on a 3-axis translation stage. Both the uniform and embossed CMUT cells were biased with the DC power supply and superposed with periodic unipolar pulses (20 Vpp, 90 ns pulse width and 100 kHz repetition frequency) provided by a waveform generator (Model: 33500B, Keysight Technologies Inc., Santa Rosa, CA, USA). The hydrophone was placed at a distance of 3 mm above the CMUTs and measured three times at each DC bias. For the reason of increasing signal-to-noise of signal [31], the peak-to-peak voltages were averaged 32 times and recorded by a digital oscilloscope (Model: DSOS254A, Keysight Technologies Inc., Santa Rosa, CA, USA). The measured pressure value *P* was converted to the unattenuated pressure on the surface of CMUT $P_{0}$ with Equation (3) in consideration of the acoustic pressure attenuation in liquid.

(3) $$P_{0}=Pe^{\beta f^{j}z},$$

where *f* depicts the frequency, *z* is the distance of acoustic wave propagating in meter, and β and *j* are the attenuation coefficients depending on the properties of liquid. In this case, β was 6.43 $\times 10^{-12}$ and *j* was 1.85 for corn oil [32].

**Figure 9:**
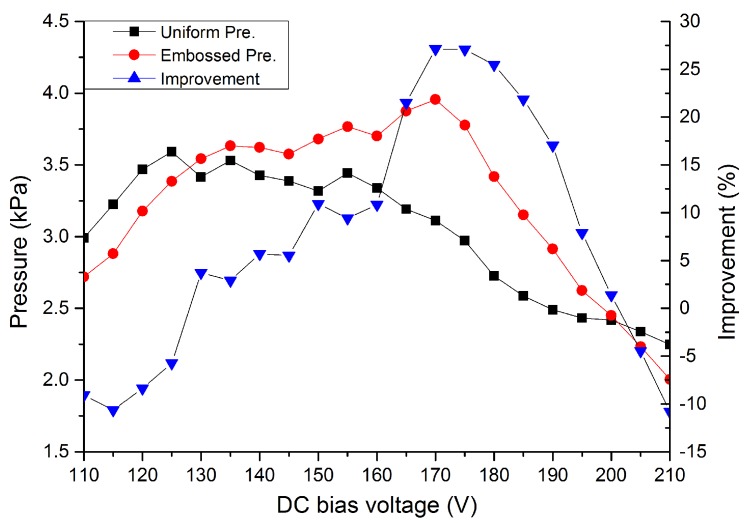
Output pressures of the uniform/embossed CMUT cells and the improvement of the embossed CMUT over uniform CMUT under different DC bias voltages.

The compensated output pressures of the embossed and uniform CMUT cells working in collapse region were plotted in Figure 9, which also depicted the pressure improvement of the embossed CMUT DC biased from 110 V to 210 V. These acoustic measurement results indicated that the embossed pattern on a collapse mode CMUT can enhance output pressure between 130 V to 200 V, which covered most of the collapse region. The pressure of the embossed CMUT was smaller than the uniform CMUT when the DC bias voltage was less than 130 V. However, with increasing bias voltage, the embossed CMUT cell owned higher pressures than the uniform CMUT cell and the improvement increased almost monotonically until a peak value of 27.1% occurred at 170 V. Then, the improvement dropped with increasing DC bias and became negative when the DC bias voltage was above 200 V. The trend of improvement with the DC bias voltage in measurement was similar to the FEA simulation as shown in Figure 3.

The position of the embossed pattern plays an important role in the pressure improvement. When it is located at the vibrating center, the structure can achieve the best performance in output pressure. However, if the embossed pattern is deviated from the vibrating center, the membrane displacement will be restricted in a way that lowers the pressure improvement, especially when the embossed pattern is close to the edge of the vibrating membrane. This is the major reason for a single peak improvement in both simulation and experiment. In this experiment, the embossed CMUT cell owned a smaller gap height and pull-in voltage than FEA simulations so that the relative operational range of the embossed pattern to achieve higher output pressures was reduced in the experiment. The improvements of pressure were negative when the DC bias voltages were less than 130 V or higher than 200 V, in which cases the embossed patterns were moved towards the outer or inner edge of the vibrating membrane that restricted the membrane vibration. Therefore, the output pressure of the embossed CMUT became lower than that of the uniform CMUT.

It should be noted that both the embossed and uniform CMUT cells were compared at the same DC bias voltage as [13,14,16,17] because it is useful for investigating the relationship between the optimal embossed pattern position and output pressure improvement. In our experiments, we aimed to measure the output pressure of the embossed CMUT for locations of embossed pattern. As it is not feasible to fabricate a series of embossed CMUTs under the same fabrication conditions, we therefore fixed the embossed pattern on the membrane and then tuned the relative position by changing the DC bias voltage. From the experiments, the maximum improvement value of 27.1% indicated that the embossed pattern was tuned to the optimal position (vibrating center) on the vibrating membrane when the embossed CMUT was biased at 170 V. Although this method is adopted from previous studies and is fitted for our initial purposes, it is not fair for the uniform CMUT cell since it was not working under its optimal operating point. Considering from this point of view, the improvement value was 10.2% by comparing the maximum values of embossed CMUT and uniform CMUT biased at 170 V and 125 V, respectively.

**Figure 10:**
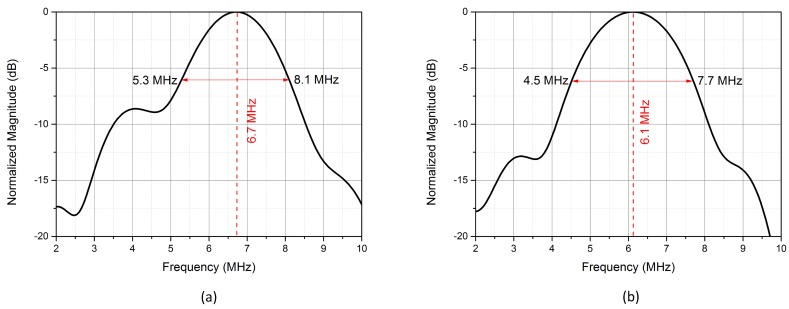
Normalized frequency spectrum of output pressures generated by (**a**) the uniform CMUT cell and (**b**) the embossed CMUT cell biased at 170 V.

Figure 10 gave the normalized frequency spectrum of pressures generated by the uniform and embossed CMUTs biased at 170 V. The center frequency of the uniform CMUT cell was 6.7 MHz, which was 0.6 MHz higher than that of the embossed CMUT cell. The 6-dB fractional bandwidths of the uniform and embossed CMUT cells were 41.8% and 52.5%, and the 6-dB pressure-bandwidth products [33] of the uniform CMUT and the embossed CMUT biased at 170 V were 8.71 kPa-MHz and 11.70 kPa-MHz, respectively. The acoustic measurements indicated that the embossed CMUT cell owned better performance in output pressure, fractional bandwidth and pressure-bandwidth product.

## 4. Discussion

**Figure 11:**
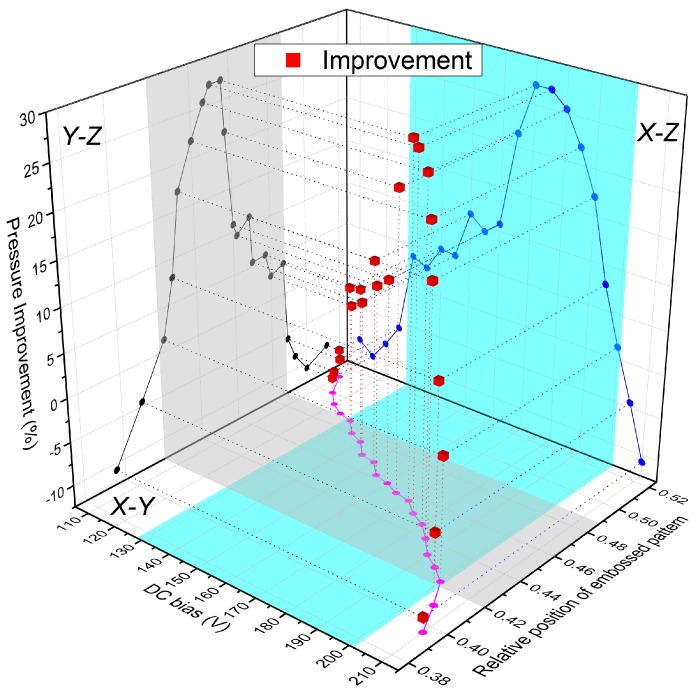
Correlation of DC bias, relative position of the embossed pattern, and pressure improvement.

It was revealed that the position of the embossed pattern was critical to the output pressure improvements in [20]. In the experiment, the contact radii of the embossed CMUT were measured under different DC bias voltages. Based on the contact radius, the relative position of the embossed pattern on vibrating membrane can also be calculated. Since the embossed pattern is centered at r= 12 μm, the relative position of the embossed pattern $R_{r}$ is defined in Equation (4).

(4) $$R_{r}=\frac{12-R_{c}}{20-Rc},$$

where $R_{c}$ is the contact radius of the membrane biased at a certain DC bias voltage. The correlation of pressure improvement, DC bias voltage, and relative position of the embossed pattern is depicted in Figure 11, in which the *x*, *y*, and *z* axes refer to DC bias voltage, relative position of the embossed pattern, and pressure improvement. The relationships of these variables were projected on the *X*–*Y*, *X*–*Z*, and *Y*–*Z* planes.

In this figure, the light blue area in the *X*–*Z* plane depicted the range of DC bias voltages in which the embossed CMUT can generate more output pressures. The gray shadow in the *Y*–*Z* plane indicated the relative positions where the embossed pattern can achieve higher pressures than that of the uniform CMUT. The overlapped area of the light blue and gray shadow in the *X*–*Y* plane represented the effective ranges of DC bias voltages and relative positions where the embossed pattern can achieve more output pressures. The maximum pressure improvement of 27.1% was achieved under 170 V DC bias voltage, in which case the relative position of the embossed pattern was 0.456. This value was less than the optimum position of 0.481 that was deduced as the vibrating center in our previous paper [20] and the deviation was 5.2%, which can be ascribed to several reasons. First, the optimum value of 0.481 was deduced from a simply supported beam model with an infinitely small embossed pattern. Due to the ability of our fabrication process, we had to fabricate a flat embossed pattern on the vibrating membrane that would blur the center of mass of the embossed pattern. Secondly, the misalignment in multiple-photolithography processes would affect the horizontal position accuracies of the embossed pattern, full top electrode, and membrane. Thirdly, the lateral resolution of the 3-D optical microscope is 0.48 μm, which also restricted the accuracies of the measured contact radii.

Although there was a deviation between the measured optimal relative position of the embossed pattern and the analytical value of 0.481, the result substantiated the conclusion that the embossed pattern should be placed at the vibrating center instead of the geometry center of the vibrating membrane for a CMUT working in collapse mode, as stated in our previous paper.

## 5. Conclusions

In this study, both the uniform and embossed CMUT cells were designed and fabricated in one die with a customized six-mask sacrificial release process. An annular embossed pattern was constructed on the full top electrode with nickel electroplating method to form an embossed CMUT. The fabricated devices were then characterized with optical, electrical, and acoustic methods. The embossed CMUT cell achieved about 27.1% higher output pressure in comparison with the uniform CMUT cell. In accordance with the analysis in reference [20], the position of an embossed pattern played a critical role on the improvement of output pressure and the maximum value can be achieved by forming an embossed pattern at the membrane vibration center. Different to simulation, the pattern was centered at r= 12 μm on a fabricated CMUT cell and it cannot be moved. However, for a CMUT working in collapse mode, the ring width of vibrating membrane is changed by exerting various DC bias voltages. Therefore, it is able to tune the membrane vibration center to the location of the embossed pattern by gradually adjusting the DC bias voltage for achieving the best improvement. In this work, both the simulation analysis and acoustic measurement showed this behavior that there was a single peak of pressure improvement at a certain DC bias voltage. It was also found that the measured optimal position of the embossed pattern biased at 170 V was close to the analytical value of the vibrating center on the membrane.

In our previous research [20], it revealed that to minimize the effect on the stiffness of membrane, the embossed pattern should be small in width and large in height. However, in this work, the width of pattern was designed as 3 μm due to some limitations in fabrication that constrained the membrane vibration and resulted in a limited improvement. In this case, the embossed CMUT cell achieved better performance than the uniform CMUT cell in output pressure, fractional bandwidth, and pressure-bandwidth product. In the future, we will explore more advance processes to fabricate an ideal embossed pattern on top electrode to further improve the performance of embossed CMUTs, and to develop the embossed and uniform CMUTs with similar pull-in voltages and/or center frequencies as well.

## Figures and Tables

**Table 1:** Design parameters of the embossed CMUT.

Dimensions	(μm)
Membrane (Si_3_N_4_) radius	20
Membrane (Si_3_N_4_) thickness	0.65
Gap height	0.30
Insulator1 (SiO_2_) thickness	0.15
Insulator2 (Si_3_N_4_) thickness	0.15
Top electrode (Gold & Chromium) radius	20
Top electrode (Gold) thickness	0.18
Top electrode (Chromium) thickness	0.02
Embossed pattern (Nickel) width	3.0
Embossed pattern (Nickel) height	2.0
Embossed pattern (Nickel) inner radius	10.5
Embossed pattern (Nickel) outer radius	13.5

## References

[1] Oralkan O., Ergun A.S., Johnson J.A., Karaman M., Demirci U., Kaviani K., Lee T.H., Khuri-Yakub B.T. (2002). Capacitive micromachined ultrasonic transducers: Next-generation arrays for acoustic imaging?. IEEE Trans. Ultrason. Ferroelectr. Freq. Control.

[2] Caronti A., Caliano G., Carotenuto R., Savoia A., Pappalardo M., Cianci E., Foglietti V. (2006). Capacitive micromachined ultrasonic transducer (CMUT) arrays for medical imaging. Microelectron. J..

[3] Butterfly Network Inc Ultrasound, Ultra-Simplified. https://www.butterflynetwork.com/.

[4] Tawfik H.H., Alsaiary T., Elsayed M.Y., Nabki F., El-Gamal M.N. (2019). Reduced-gap CMUT implementation in PolyMUMPs for air-coupled and underwater applications. Sens. Actuators A Phys..

[5] Park S., Yoon I., Lee S., Kim H., Seo J.-W., Chung Y., Unger A., Kupnik M., Lee H.L. (2018). CMUT-based resonant gas sensor array for VOC detection with low operating voltage. Sens. Actuators B Chem..

[6] Pun S.H., Yu Y., Zhang J., Wang J., Cheng C.-H., Lei K.F., Yuan Z., Mak P.U. (2018). Monolithic Multiband CMUTs for Photoacoustic Computed Tomography With In Vivo Biological Tissue Imaging. IEEE Trans. Ultrason. Ferroelectr. Freq. Control.

[7] Brenner K., Ergun A.S., Firouzi K., Rasmussen M.F., Stedman Q., Khuri–Yakub B.P. (2019). Advances in Capacitive Micromachined Ultrasonic Transducers. Micromachines.

[8] Huang Y., Zhuang X., Haeggstrom E.O., Ergun A.S., Cheng C.-H., Khuri-Yakub B.T. (2009). Capacitive micromachined ultrasonic transducers with piston-shaped membranes: fabrication and experimental characterization. IEEE Trans. Ultrason. Ferroelectr. Freq. Control.

[9] Guldiken R.O., Zahorian J., Yamaner F.Y., Degertekin F.-L. (2009). Dual-electrode CMUT with non-uniform membranes for high electromechanical coupling coefficient and high bandwidth operation. IEEE Trans. Ultrason. Ferroelectr. Freq. Control.

[10] Kim D.K., Chung S.-W., Jeong B.-G., Hong S.-W., Shin H. (2013). An indirectly clamped capacitive micromachined ultrasonic transducer with a high electromechanical coupling factor. Sens. Actuators A Phys..

[11] Emadi T.A., Buchanan D.A. (2015). A novel 6 × 6 element MEMS capacitive ultrasonic transducer with multiple moving membranes for high performance imaging applications. Sens. Actuators A Phys..

[12] Lee B.C., Nikoozadeh A., Park K.K., Khuri-Yakub B.T. (2018). High-Efficiency Output Pressure Performance Using Capacitive Micromachined Ultrasonic Transducers with Substrate-Embedded Springs. Sensors.

[13] Na S., Li Z., Wong L.L., Chen A.I.-H., Macecek M., Yeow J. (2017). An optimization and comparative study of air-coupled CMUT cells with circular and annular geometries. IEEE Trans. Ultrason. Ferroelectr. Freq. Control.

[14] Na S., Chen A.I., Wong L.L., Li Z., Macecek M., Yeow J. (2016). Capacitive micromachined ultrasonic transducers based on annular cell geometry for air-coupled applications. Ultrasonics.

[15] Shiwei Z., Reynolds P., Hossack J.A. (2005). Improving the performance of capacitive micromachined ultrasound transducers using modified membrane and support structures. IEEE Ultrason. Symp..

[16] Park K.K., Oralkan O., Khuri-Yakub B.T. (2013). A comparison between conventional and collapse-mode capacitive micromachined ultrasonic transducers in 10-MHz 1-D arrays. IEEE Trans. Ultrason. Ferroelectr. Freq. Control.

[17] Huang Y., Haegstrom E., Bayram B., Zhuang X., Ergun A.S., Cheng C., Khuri-Yakub B.T. (2006). Comparison of conventional and collapsed region operation of capacitive micromachined ultrasonic transducers. IEEE Trans. Ultrason. Ferroelectr. Freq. Control.

[18] Bayram B., Oralkan O., Ergun A.S., Haeggstrom E., Yaralioglu G.G., Khuri-Yakub B.T. (2005). Capacitive micromachined ultrasonic transducer design for high power transmission. IEEE Trans. Ultrason. Ferroelectr. Freq. Control.

[19] Olcum S., Yamaner F.Y., Bozkurt A., Atalar A. (2011). Deep-collapse operation of capacitive micromachined ultrasonic transducers. IEEE Trans. Ultrason. Ferroelectr. Freq. Control.

[20] Yu Y., Pun S.H., Mak P.U., Cheng C.-H., Wang J., Mak P.-I., Vai M.I. (2016). Design of a Collapse-Mode CMUT with an Embossed Membrane for Improving Output Pressure. IEEE Trans. Ultrason. Ferroelectr. Freq. Control.

[21] Yu Y., Wang J., Pun S.H., Cheng C.-H., Lei K.F., Vai M.I., Zhang S., Mak P.U. (2019). Fabrication of embossed capacitive micromachined ultrasonic transducers using sacrificial release process. IEICE Electron. Express.

[22] Zhuang X. (2008). Capacitive Micromachined Ultrasonic Transducers with Through-Wafer Interconnects. Ph.D. Thesis.

[23] Coupland J.N., McClements D.J. (1997). Physical properties of liquid edible oils. J. Am. Oil Chem. Soc..

[24] Li Z., Zhao L., Ye Z., Wang H., Zhao Y., Jiang Z. (2013). Resonant frequency analysis on an electrostatically actuated microplate under uniform hydrostatic pressure. J. Phys. D Appl. Phys..

[25] Li Z., Zhao L., Jiang Z., Ye Z., Zhao Y. (2015). An improved method for the mechanical behavior analysis of electrostatically actuated microplates under uniform hydrostatic pressure. J. Microelectromech. Syst..

[26] Yaralioglu G.G., Ergun S.A., Khuri-Yakub B.T. (2005). Finite-element analysis of capacitive micromachined ultrasonic transducers. IEEE Trans. Ultrason. Ferroelectr. Freq. Control.

[27] Crisfield M.A. (1991). Non-Linear Finite Element Analysis of Solids and Structures.

[28] Hadian S., Gabe D. (1999). Residual stresses in electrodeposits of nickel and nickel–iron alloys. Surf. Coat. Technol..

[29] George A., Di B. (2011). Modern Electroplating.

[30] Luo J.K., Pritschow M., Flewitt A.J., Spearing S.M., Fleck N.A., Milne W.I. (2006). Effects of process conditions on properties of electroplated Ni thin films for microsystem applications. J. Electrochem. Soc..

[31] Cetin A.M., Bayram B. (2013). Diamond-based capacitive micromachined ultrasonic transducers in immersion. IEEE Trans. Ultrason. Ferroelectr. Freq. Control.

[32] Chanamai R., McClements D.J. (1998). Ultrasonic attenuation of edible oils. J. Am. Oil Chem. Soc..

[33] Olcum S., Senlik M.N., Atalar A. (2005). Optimization of the gain-bandwidth product of capacitive micromachined ultrasonic transducers. IEEE Trans. Ultrason. Ferroelectr. Freq. Control.

